# Supplementary material for: Synthesis of length-tunable DNA carriers for nanopore sensing
Source: PLoS One. 2023 Aug 23;18(8):e0290559. doi: 10.1371/journal.pone.0290559 (PMC10446168; doi:10.1371/journal.pone.0290559)
Supplement: S1 File — (PDF) [file pone.0290559.s001.pdf]

S1 Section: Oligonucleotide sequences

Table S1: *List of oligonucleotide sequences used to create the sticky-ended DNA products in the main text, using λ-DNA as a PCR template. All oligos were ordered without a 5' terminal phosphate group, especially important in the case of the linker sequences for preventing the permanent formation of circles/concatemers during the ligation step (see Fig. 1a in the main text). Bases highlighted in red and green represent sequences that will eventually constitute (3') overhangs that are compatible with those from digestion with KpnI and SacI restriction enzymes, respectively. In particular, the bolded adenine in the forward primer for the 1.8-kbp product represents a base that differs from the native sequence of λ-DNA and is used to introduce a SacI recognition site in the PCR amplicon. Finally, the underlined sequences in the linker oligos represent regions of complementarity used to hybridize the two strands into an assembled linker molecule.*

**Category**	**Name**	**Sequence**
1.8-kbp Primers	'FWD-1.8kbp'	CAGTATGG**A**GCTCGGTGGTGTG
	'RVS-1.8kbp'	AGGAACACGGCTCACTTTTACCTT
6.2-kbp Primers	'FWD-6.2kbp'	GGGACGCTCAGTAATGTGACGATA
	'RVS-6.2kbp'	GGAACTCCGGGTGCTATCAGTTTT
Linkers	'cosL-KpnI'	GGGCGGCGACCTGTAC
	'cosR-SacI'	AGGTCGCCGCCCAGCT
