# Supplementary material for: Synthesis of length-tunable DNA carriers for nanopore sensing
Source: PLoS One. 2023 Aug 23;18(8):e0290559. doi: 10.1371/journal.pone.0290559 (PMC10446168; doi:10.1371/journal.pone.0290559)
Supplement: S2 File — (PDF) [file pone.0290559.s002.pdf]

S2 Section: Detailed SE-DNA synthesis protocols

A) Polymerase Chain Reaction

1. Combine on ice:
   - 3 µL dNTPs (10 mM, New England Biolabs)
   - 4 µL forward primer (10 µM, Integrated DNA Technologies)
   - 4 µL reverse primer (10 µM, IDT)
   - 1 µL λ-DNA template (20 ng/µL, NEB)
   - 4 µL LongAmp Taq polymerase (2.5 U/µL, NEB)
   - 20 µL LongAmp reaction buffer (5×, NEB)
   - 64 µL nuclease-free water (Invitrogen)

2. Place in thermocycler:

Time	Temperature	Cycles
30 s	94 °C	×1
15 s	94 °C	
30 s	60 °C	×30
5 min 20 s	65 °C	
10 min	65 °C	×1

3. Recover DNA product with spin column (e.g. PureLink PCR Purification Kit, Invitrogen) per manufacturer's instructions

B) Double Restriction Digest

1. Combine on ice:
   - 16 µL PCR product (~500 ng/µL, from reaction A)
   - 2 µL KpnI-HF restriction enzyme (20 U/µL, NEB)
   - 2 µL SacI-HF restriction enzyme (20 U/µL, NEB)
   - 10 µL CutSmart reaction buffer (10×, NEB)
   - 70 µL nuclease-free water (Invitrogen)

2. Incubate at 37 °C for 1+ hrs

3. Recover DNA product with spin column (e.g. PureLink PCR Purification Kit, Invitrogen) per manufacturer's instructions

C) <u>Linker Ligation</u>

1. Anneal linker strands by combining 1 µL linker oligo 1 (100 µM, IDT), 1 µL linker oligo 2 (100 µM, IDT), 1 µL T4 ligase reaction buffer (10×, Thermo Scientific), and 7 µL nuclease-free water (Invitrogen) in a tube and then heating for 2 min at 95 °C, 10 min at 50 °C, and cooling to room temperature.

2. Combine on ice:
    - 55 µL digested DNA (~70 ng/µL, from reaction B)
    - 10 µL annealed linkers (10 µM, from step 1)
    - 4 µL T4 ligase (5 U/µL, Thermo Scientific)
    - 19 µL T4 ligase reaction buffer (10×, Thermo Scientific)
    - 112 µL nuclease-free water (Invitrogen)

3. Incubate overnight at 16 °C

4. Recover DNA product with spin column (e.g. PureLink PCR Purification Kit, Invitrogen) per manufacturer's instructions
