# Supplementary material for: Synthesis of length-tunable DNA carriers for nanopore sensing
Source: PLoS One. 2023 Aug 23;18(8):e0290559. doi: 10.1371/journal.pone.0290559 (PMC10446168; doi:10.1371/journal.pone.0290559)
Supplement: S3 File — (PDF) [file pone.0290559.s003.pdf]

S3 Section: Sample distribution of product lengths

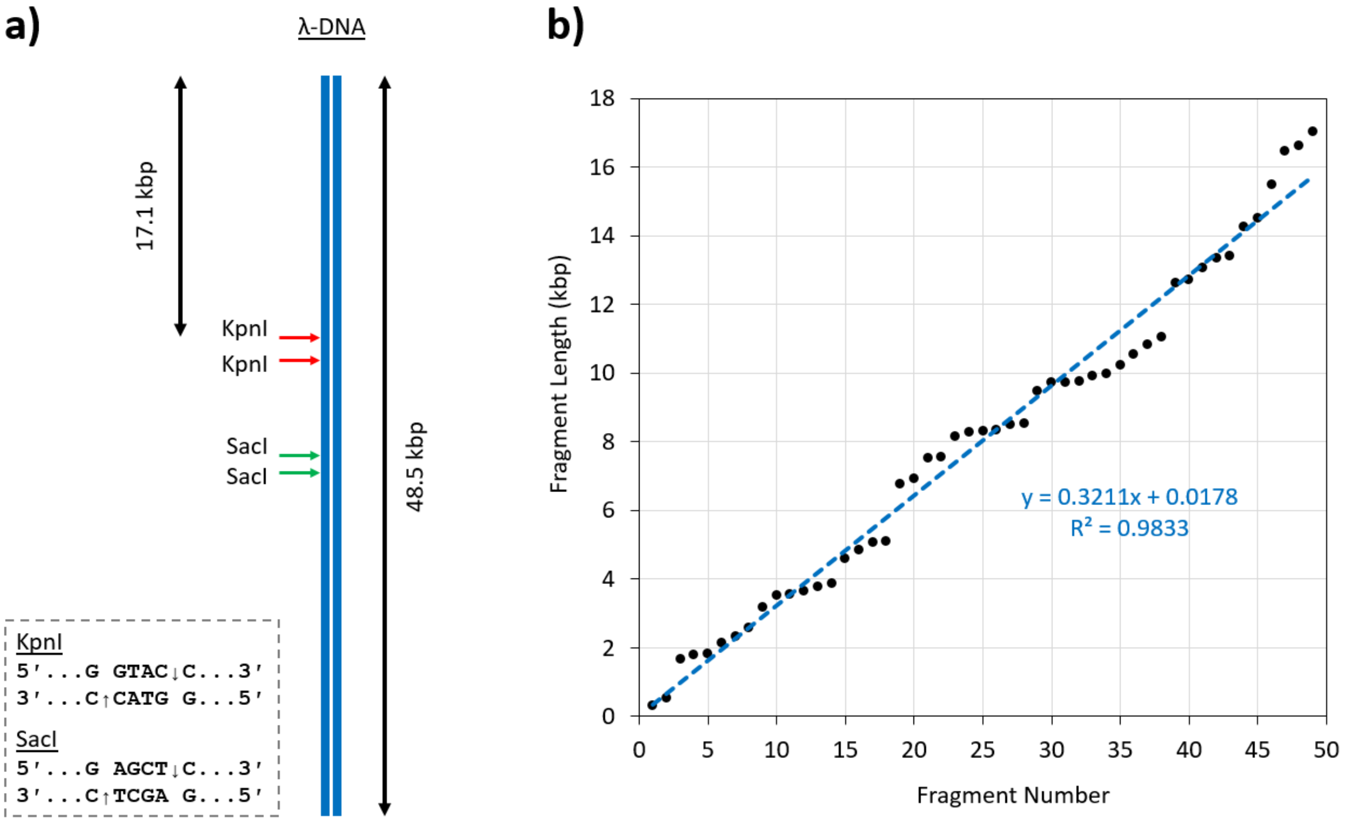

Figure S3: a) *Restriction map of λ-DNA for KpnI (red arrows) and SacI (green arrows) restriction enzymes. Each enzyme has only two (relatively closely-spaced) recognition sites in the entire 48.5 kbp template – see inset for sequences. Labelled here in particular is an initial stretch of 17.1 kbp that is devoid of recognition sites for either enzyme.* ***b)*** *Distribution of possible fragment sizes within this 17.1 kbp region by digest at: 1) the KpnI site bounding the region and 2) new SacI sites introduced by modification of the native λ sequence by a single base, using a mismatched PCR primer (e.g. 'FWD-1.8kbp' in Table S1). A full range of relatively equally-spaced (median gap size of 227 bp) fragments is produced from 0 – 17 kbp, where each fragment will feature two different overhang sequences on their ends (from SacI and KpnI – both 3' polarity, 4-nt) for selective targeting by a linker molecule in the ligation step (see Fig. 1a in the main text). Note that the density of fragment size coverage can be increased (essentially arbitrarily) beyond that of this simple example by loosening the degree of complementarity between the primer and the template (especially by simply adding the SacI recognition sequence to the upstream / 5' end of the primer), or by simultaneously adjusting the location of the KpnI site in a similar manner.*
