# Supplementary material for: Synthesis of length-tunable DNA carriers for nanopore sensing
Source: PLoS One. 2023 Aug 23;18(8):e0290559. doi: 10.1371/journal.pone.0290559 (PMC10446168; doi:10.1371/journal.pone.0290559)
Supplement: S4 File — (PDF) [file pone.0290559.s004.pdf]

S4 Section: Agarose gels of short fragment removal

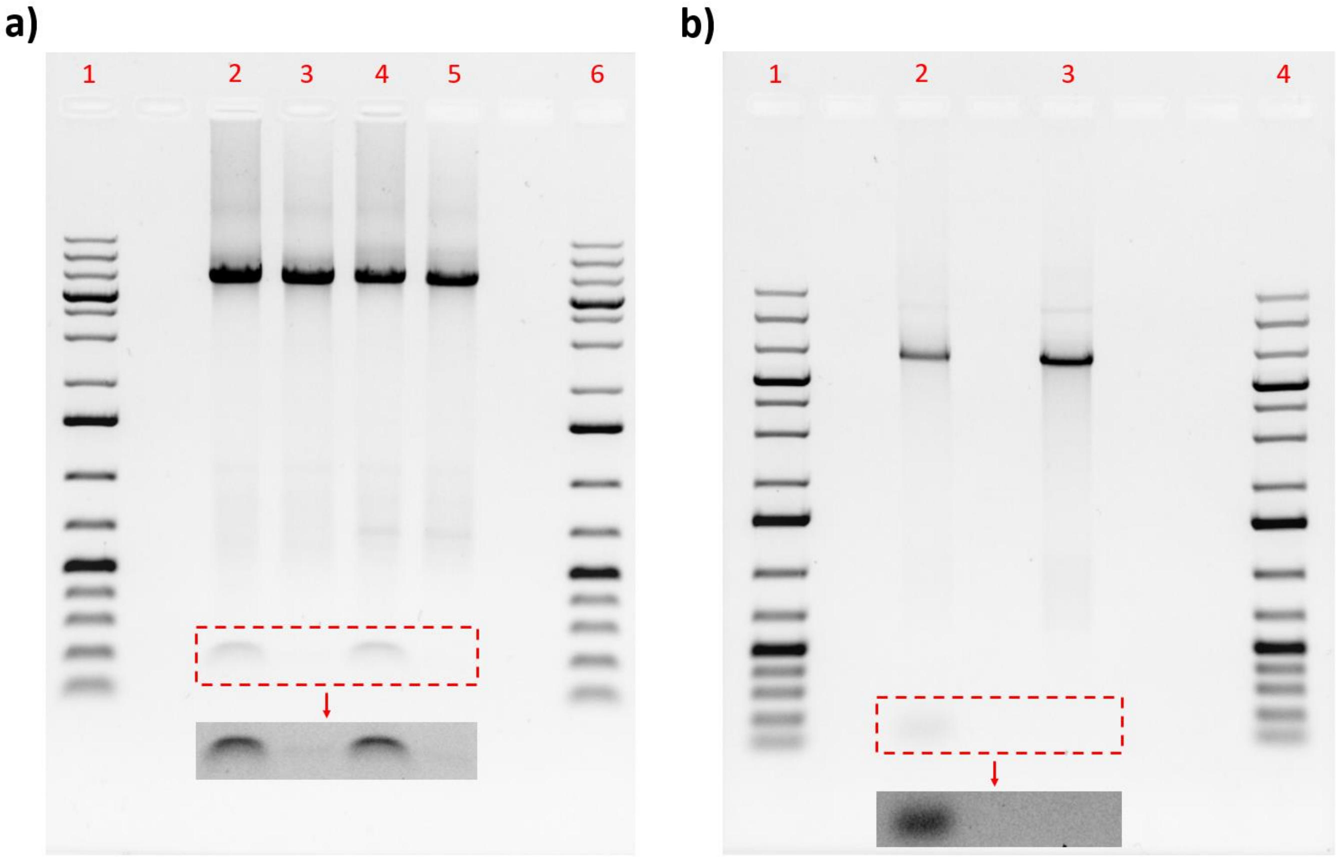

Figure S4: *Removing short spurious fragments during SE-DNA production by spin column purification.* ***a)*** *Agarose gel (0.7%, 0.5× TBE, pre-stained with Gel Red, 70 V) of KpnI-SacI double digest of 6.2 kbp PCR amplicon. Lanes 1 & 6: GeneRuler 1 kb Plus DNA Ladder (Thermo Scientific). Lanes 2 & 4: raw KpnI-SacI digest mix with 6.2 kbp amplicon from two alternate primer sets. Lanes 3 & 5: spin column purification (PureLink Quick PCR Purification Kit, Invitrogen) of Lane 2 and Lane 4 products, respectively. An enhanced-contrast view of the dashed region is presented as an inset, where the sticky-ended "caps" (70 and 144 bp or 49 and 144 bp) of the central digest fragment (see Fig 1a in the main text) are observed to have been largely removed during the purification step.* ***b)*** *Agarose gel (0.6%, 0.5× TAE, pre-stained with Gel Red, 70 V) of T4 ligation of the 6.2 kbp double-digest product from (a) and a "linker" molecule (sequence in Table S1). Lanes 1 & 4: GeneRuler 1 kb Plus DNA Ladder (Thermo Scientific). Lane 2: raw T4 ligation mix. Lane 3: spin column purification (PureLink Quick PCR Purification Kit, Invitrogen) of Lane 2 products. An enhanced-contrast view of the dashed region again shows the removal of short spurious DNA fragments, this time of excess linkers (16 bp). By keeping the DNA by-products produced/present at each synthesis stage short in length (and thus easily removable) though careful sequence design, they will not be available to interfere in subsequent synthesis stages by re-annealing with the main product.*
