# Supplementary material for: Synthesis of length-tunable DNA carriers for nanopore sensing
Source: PLoS One. 2023 Aug 23;18(8):e0290559. doi: 10.1371/journal.pone.0290559 (PMC10446168; doi:10.1371/journal.pone.0290559)
Supplement: S5 File — (PDF) [file pone.0290559.s005.pdf]

S5 Section: Nanopore sensing of agarose-extracted DNA

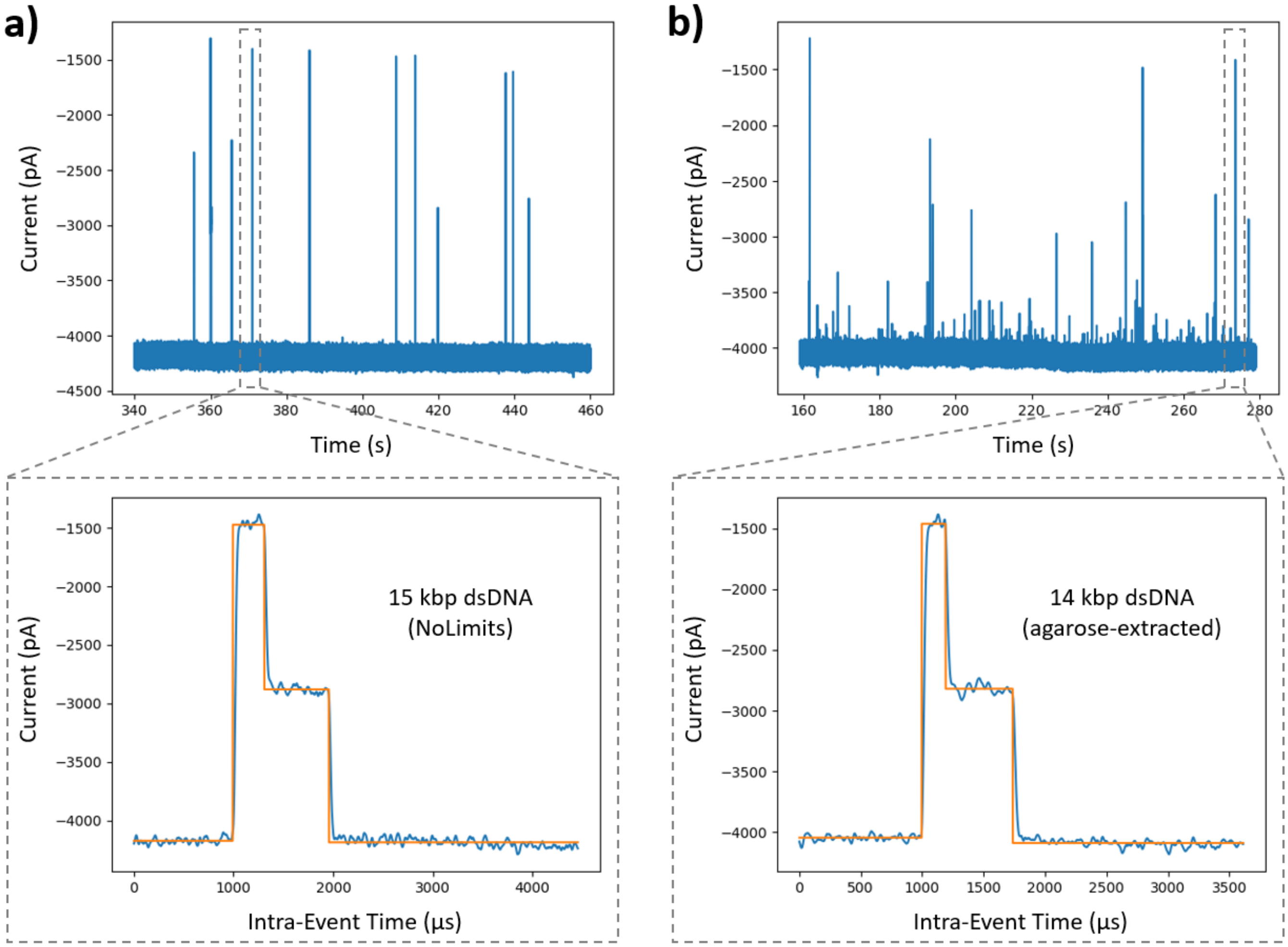

Figure S5: *Comparing nanopore signals (~4.9-nm pore diameter, 200 mV transmembrane potential, 3.6 M LiCl pH 8 buffer) produced by commercial and agarose-extracted DNA samples.* ***a)*** *Representative (2 minute) current trace of a 15 kbp commercial dsDNA sample (NoLimits, Thermo Scientific) passing through the pore.* ***b)*** *Representative (2 minute) current trace of a 14 kbp dsDNA sample (BstEII digest fragment of λ-DNA) extracted from an agarose gel (GeneJET Gel Extraction Kit, Thermo Scientific) passing through the same pore as in (a). While both samples produced recognizable dsDNA signals (example traces expanded as insets), agarose-extracted DNA reliably led to the appearance of additional, lower amplitude signals (see S5b) as if from contaminants, as well as increased pore clogging (both reversible and irreversible with voltage inversion or solution flushing). This behaviour persisted across all of the numerous extraction methods tested including gel dissolution (e.g. QIAquick, QIAGEN), centrifugal separation (e.g. Freeze 'N Squeeze, Bio-Rad), enzymatic digestion (e.g. agarase, NEB), and passive diffusion into supernatant out of submerged gel pieces. In contrast, DNA synthesis protocols that avoided an agarose-extraction step (but with otherwise similar sample manipulations), produced molecules that resulted in longer-lasting, cleaner-to-analyze nanopore experiments (such as presented in Figure 2 of the main text). Alternative approaches to isolating specific DNA fragments were thus implemented in this work (see Section S4).*
