# Supplementary material for: Synthesis of length-tunable DNA carriers for nanopore sensing
Source: PLoS One. 2023 Aug 23;18(8):e0290559. doi: 10.1371/journal.pone.0290559 (PMC10446168; doi:10.1371/journal.pone.0290559)
Supplement: S6 File — (PDF) [file pone.0290559.s006.pdf]

S6 Section: Agarose gel of carrier/DNA star hybridization

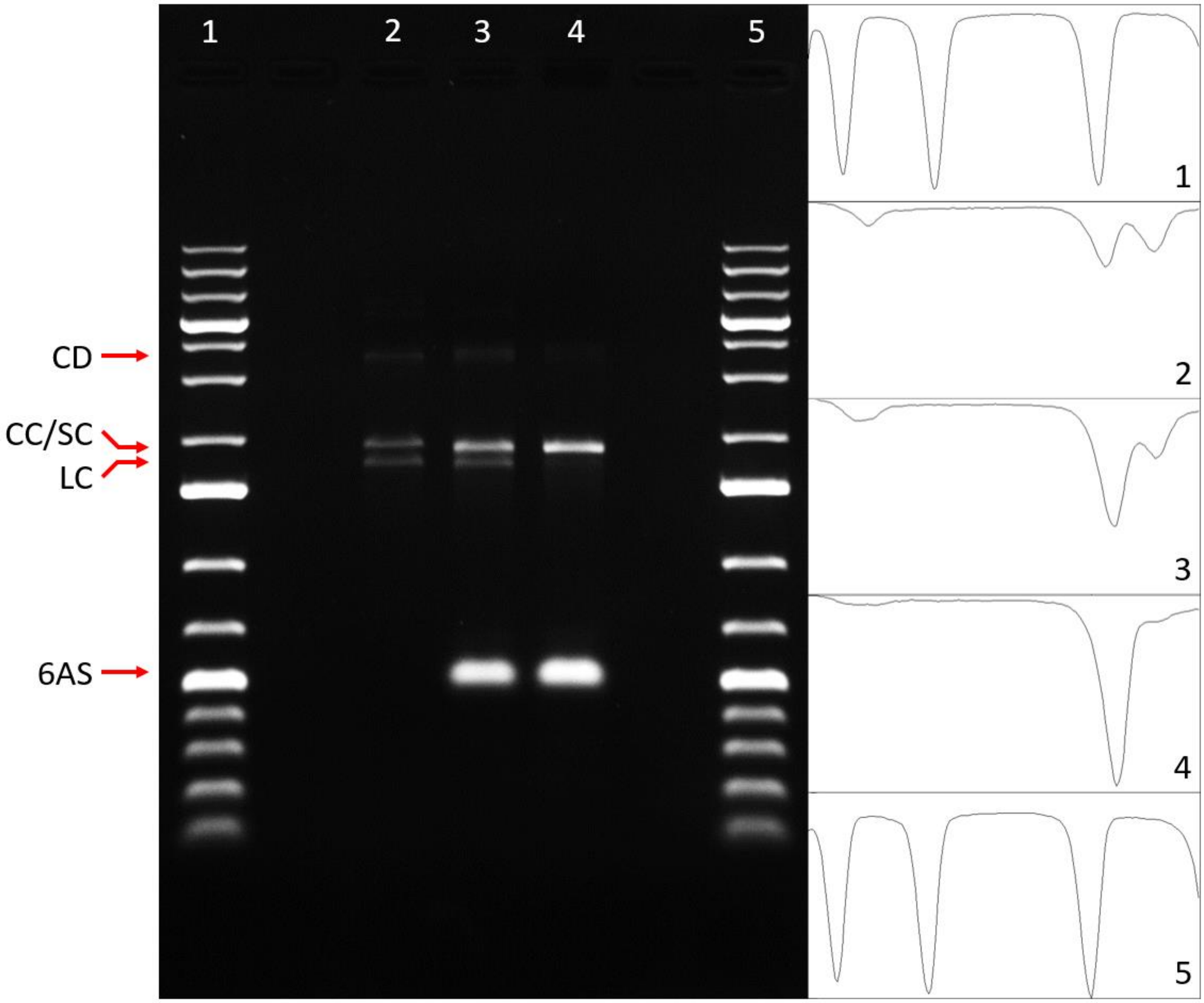

Figure S6: *Agarose gel (0.7%, 0.5× TBE, pre-stained with Gel Red, 70 V) showing the hybridization of 1.8-kbp carriers to 6-arm stars via complementary single-stranded extensions on both pieces. Lanes 1 & 5: GeneRuler 1 kb Plus DNA Ladder (Thermo Scientific). Lane 2: 1.8-kbp SE-DNA incubated for 1 hr at 37 °C (10 mM $MgCl_2$ + 10 mM Tris, pH 8). Lane 3: 1.8-kbp SE-DNA incubated with 6-arm stars (at ~100× excess) for 1 hr at 37 °C. Lane 4: 1.8-kbp SE-DNA heated 10 min at 70 °C, flash chilled, then incubated with 6-arm stars (at ~100× excess) for 1 hr at 37 °C. Four main bands are visible across the product lanes and are labelled "6AS" (6-arm stars), "LC" (linear carriers), "CC" (circular carriers), "SC" (star-annealed carriers), and "CD" (carrier dimers) after their presumptive molecular identities. The panels on the right show the integrated band intensities at the positions of the top three (carrier-associated) bands for each lane. As the same mass of carrier was loaded into all product lanes, the increase in intensity of the CC/SC band in lane 3 relative to lane 2 (lacking DNA stars) is attributed to the attachment of stars to a portion of the carriers. The further intensity increase of this band in lane 4 (combined with the simultaneous reduction in the LC band) when the star incubation was preceded by heating the carriers above the melting temperature of their ends implies that some fraction of the carriers were inaccessible to the stars (e.g. circularized or attached to stray linker oligos) prior to this melting step. Such a step was therefore incorporated into subsequent annealing reactions when producing analytes for nanopore sensing.*
