# Supplementary material for: Synthesis of length-tunable DNA carriers for nanopore sensing
Source: PLoS One. 2023 Aug 23;18(8):e0290559. doi: 10.1371/journal.pone.0290559 (PMC10446168; doi:10.1371/journal.pone.0290559)
Supplement: S7 File — (PDF) [file pone.0290559.s007.pdf]

S7 Section: Filtering out short-lived nanopore events

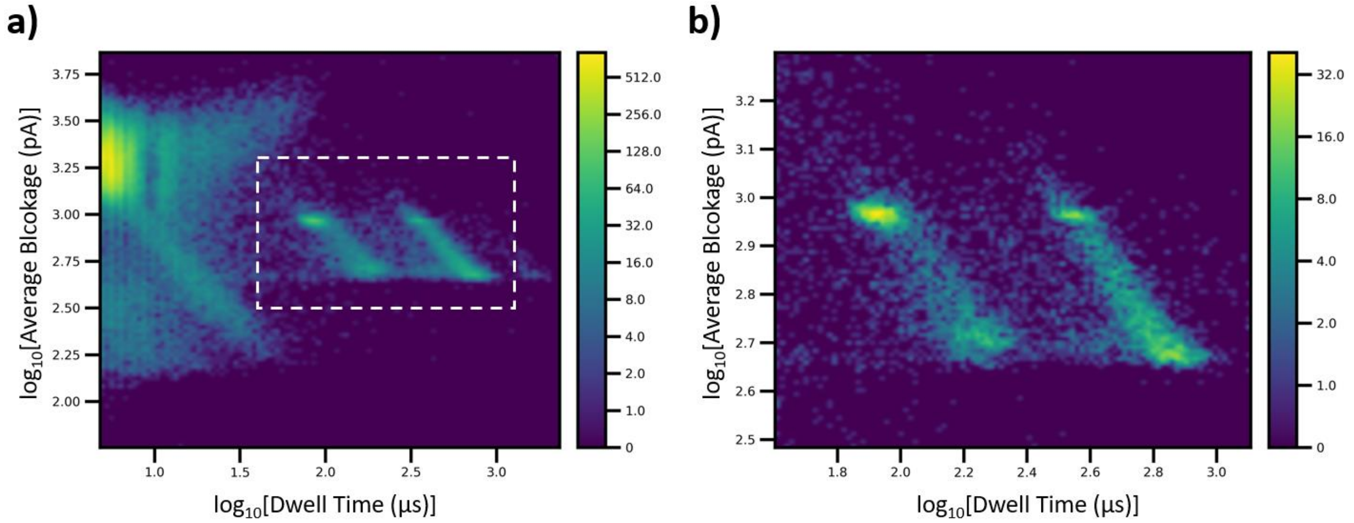

Figure S7: a) *2D histogram of average blockage vs. dwell time (log-log scaling) for all nanopore events from the experiment represented in Figure 2 of the main text (1.8- and 6.2-kbp DNA carriers, 12-arm stars run on ~13 nm pore in 3.6 M LiCl pH 8 buffer, 150 mV transmembrane potential). Two main populations of long-lived events are visible on the right side of the plot, corresponding to translocations of the two carrier sizes (attached to a DNA star or otherwise). This contrasts with the diffuse mass of short-lived events on the left side of the plot, widely-spread in average blockage, which likely correspond to failed pore entries (“collisions”) as well as to translocations of free 12-arm stars (added in large excess to the carriers), especially those events with the deepest average blockages. A set of event selection filters, represented by the dashed box in S7a, was applied to the data to only consider events from the carriers during analysis: dwell time ∈ [40 µs, 1300 µs] & average blockage ∈ [300 pA, 2000 pA].* ***b)*** *2D histogram of average blockage vs. dwell time (log-log scaling) for this filtered set of events, showing the isolation of the two main carrier populations. This reduced subset was then used for the analysis of carrier translocations shown in Figure 2 of the main text.*
