# Supplementary material for: Synthesis of length-tunable DNA carriers for nanopore sensing
Source: PLoS One. 2023 Aug 23;18(8):e0290559. doi: 10.1371/journal.pone.0290559 (PMC10446168; doi:10.1371/journal.pone.0290559)
Supplement: S8 File — (PDF) [file pone.0290559.s008.pdf]

S8 Section: Average blockages of Fig 2 populations

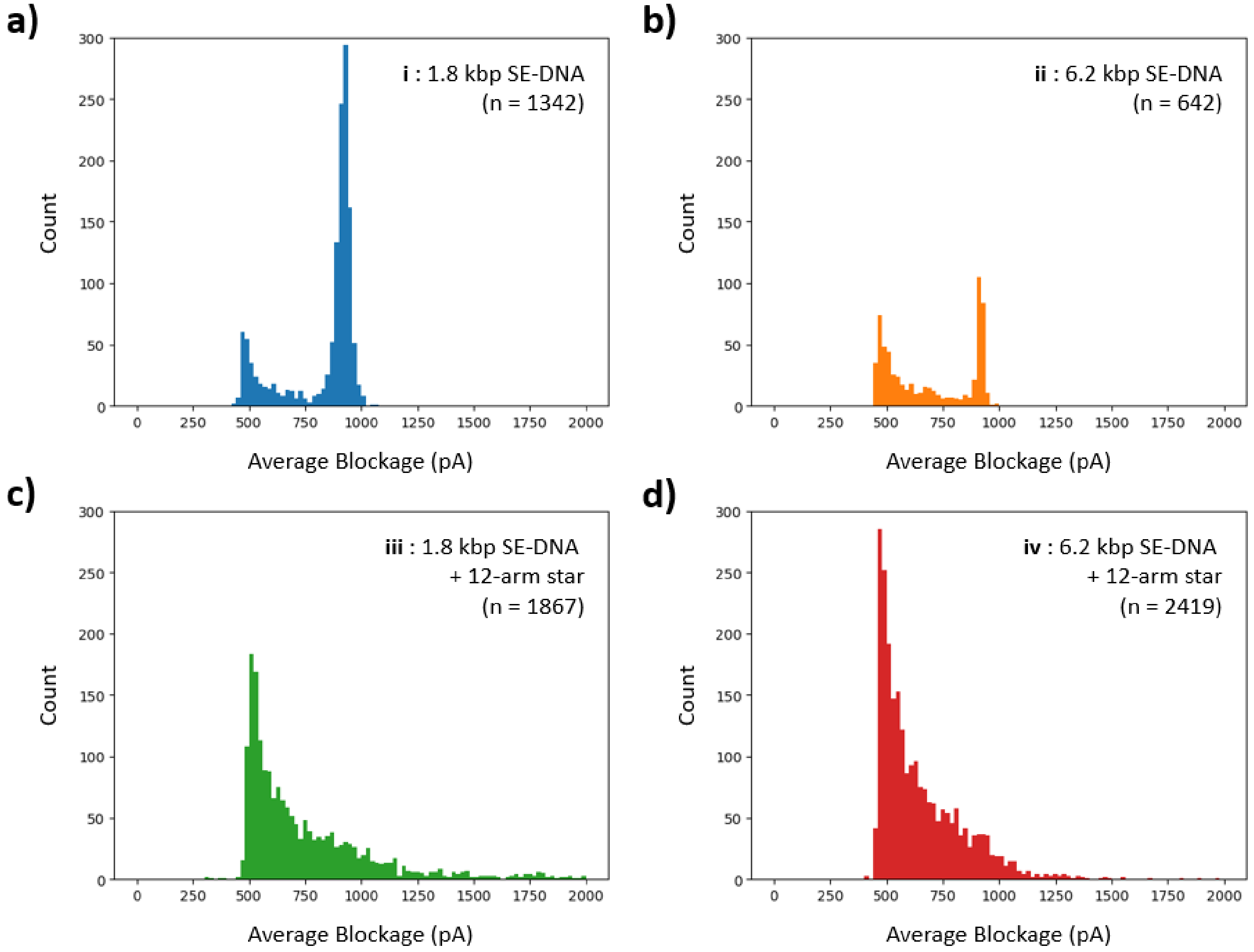

Figure S8: *1D histograms of average blockage for the events of Figure 2 in the main text (1.8- and 6.2-kbp DNA carriers, 12-arm stars run on ~13 nm pore in 3.6 M LiCl pH 8 buffer, 150 mV transmembrane potential), separated by the population types (i, ii, iii, iv) of Figure 2. The events were sorted by population using selection filters as outlined in Fig. S7, this time on max deviation and truncated ECD.* ***a)*** *Average blockage distribution of population i (1.8 kbp SE-DNA).* ***b)*** *Average blockage distribution of population ii (6.2 kbp SE-DNA).* ***c)*** *Average blockage distribution of population iii (1.8 kbp SE-DNA annealed to 12-arm star).* ***d)*** *Average blockage distribution of population iv (6.2 kbp SE-DNA annealed to 12-arm star). The star-attached populations (iii, iv) show a slow decay of average blockages from the single dsDNA level (~450 pA, fully unfolded events) to the double dsDNA level (~900 pA, fully folded events) as these molecules get captured closer and closer to the middle of their contour lengths. The free carrier populations (i, ii), on the other hand, show sharp peaks at the double dsDNA level, implying that an elevated proportion translocate in a fully-folded conformation. This is consistent with many of the free carriers having their ends annealed together to form a closed circle – this forces the molecules to pass through the pore two dsDNA fragments at a time and blocks their annealing to complementary target stars.*
