# Supplementary material for: Synthesis of length-tunable DNA carriers for nanopore sensing
Source: PLoS One. 2023 Aug 23;18(8):e0290559. doi: 10.1371/journal.pone.0290559 (PMC10446168; doi:10.1371/journal.pone.0290559)
Supplement: S9 File — (PDF) [file pone.0290559.s009.pdf]

**S9 Section: Raw gel images**

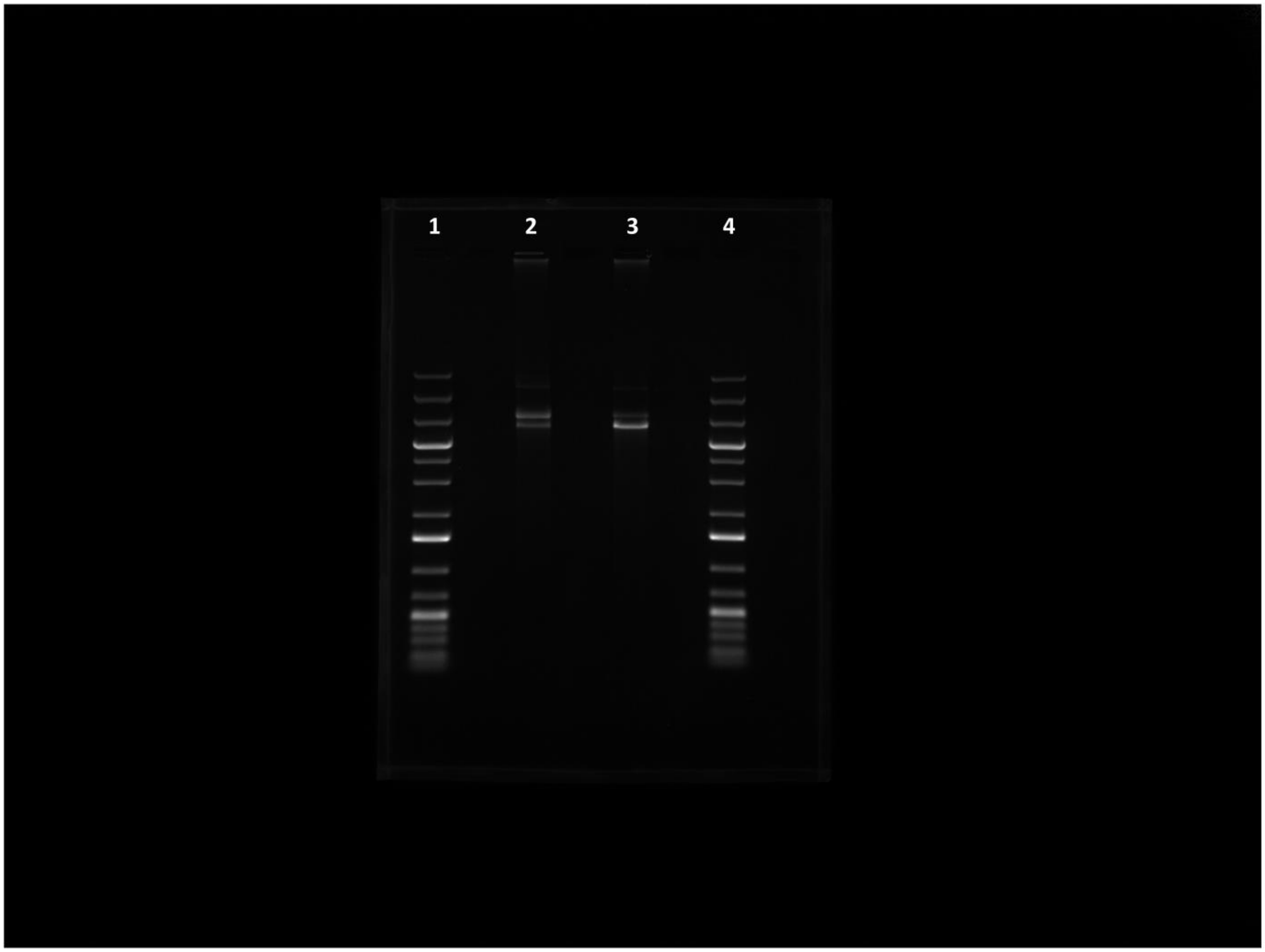


***Figure S9-1:*** *Unprocessed agarose gel image (0.5% agarose, 0.5× TAE, stained with 1× Gel Red, run at 70 V) used to create Fig 1b in the main text, with loaded lanes enumerated. Full details of each loaded sample are available in the caption there (Fig 1b, main text).*

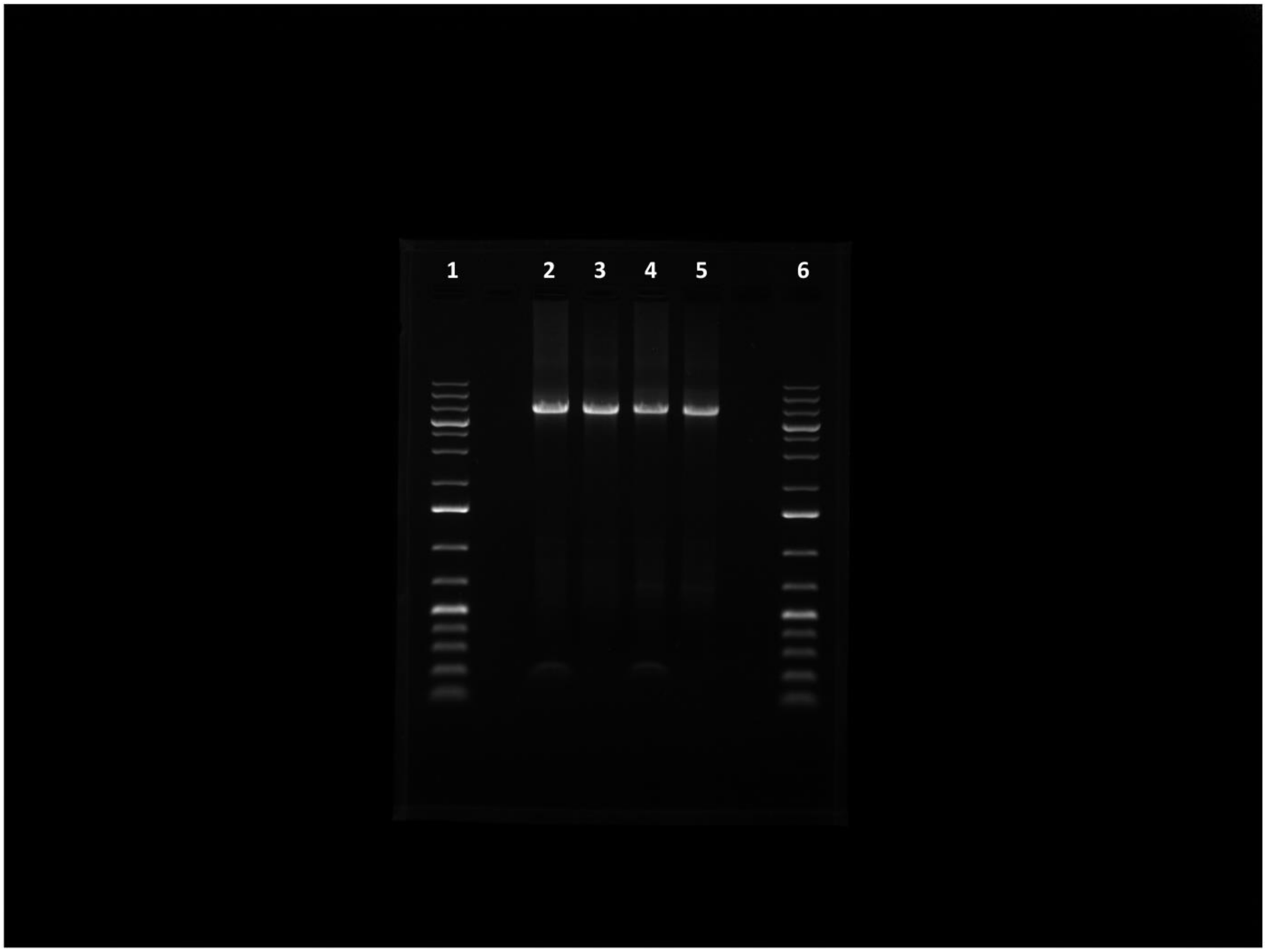


***Figure S9-2:*** *Unprocessed agarose gel image (0.7% agarose, 0.5× TBE, stained with 1× Gel Red, run at 70 V) used to create Figure S4a in the S4 Section of the Supporting Information, with loaded lanes enumerated. Full details of each loaded sample are available in the caption there (Figure S4a, S4 Section).*

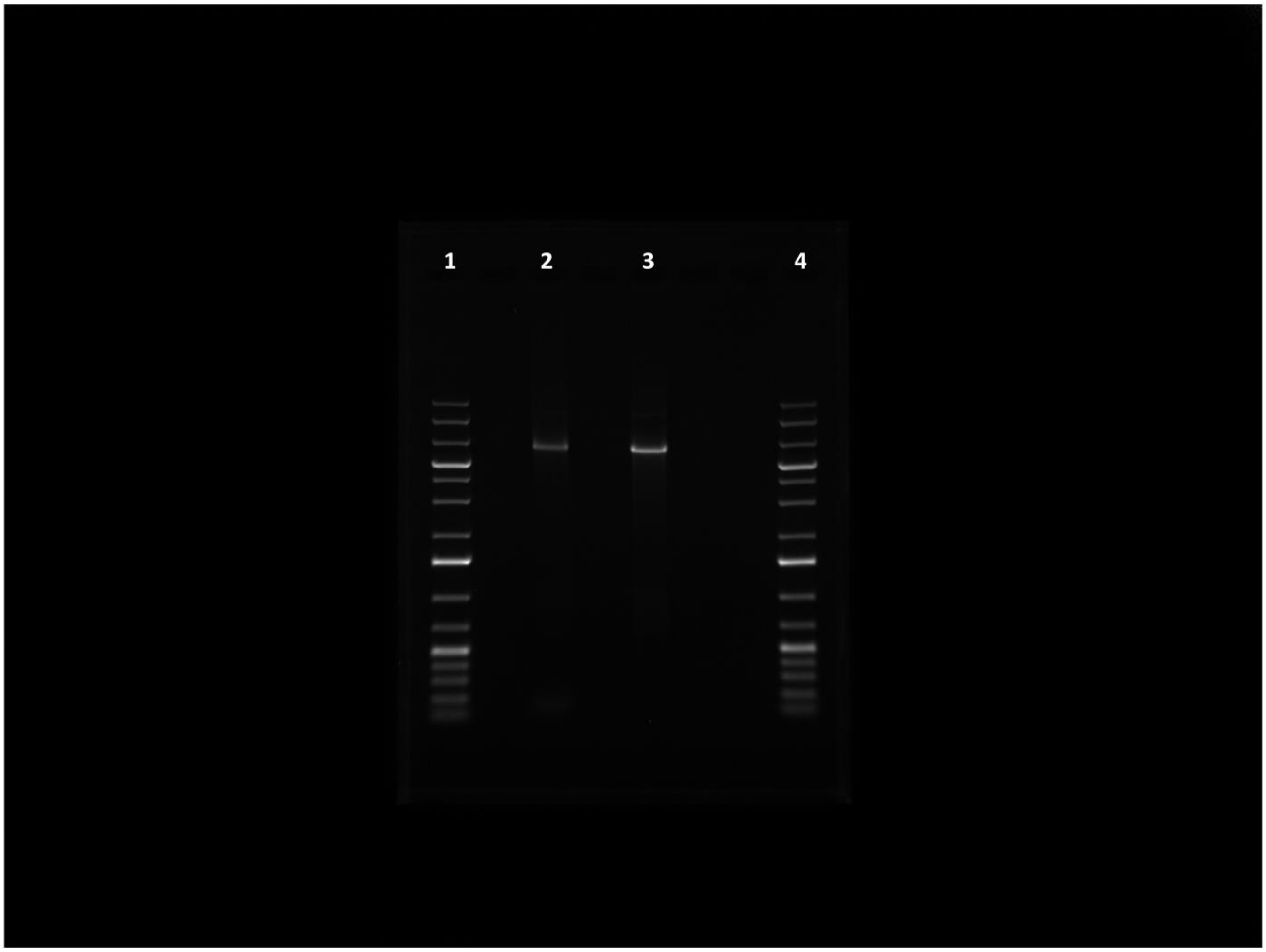


***Figure S9-3:*** *Unprocessed agarose gel image (0.6% agarose, 0.5× TAE, stained with 1× Gel Red, run at 70 V) used to create Figure S4b in the S4 Section of the Supporting Information, with loaded lanes enumerated. Full details of each loaded sample are available in the caption there (Figure S4b, S4 Section).*

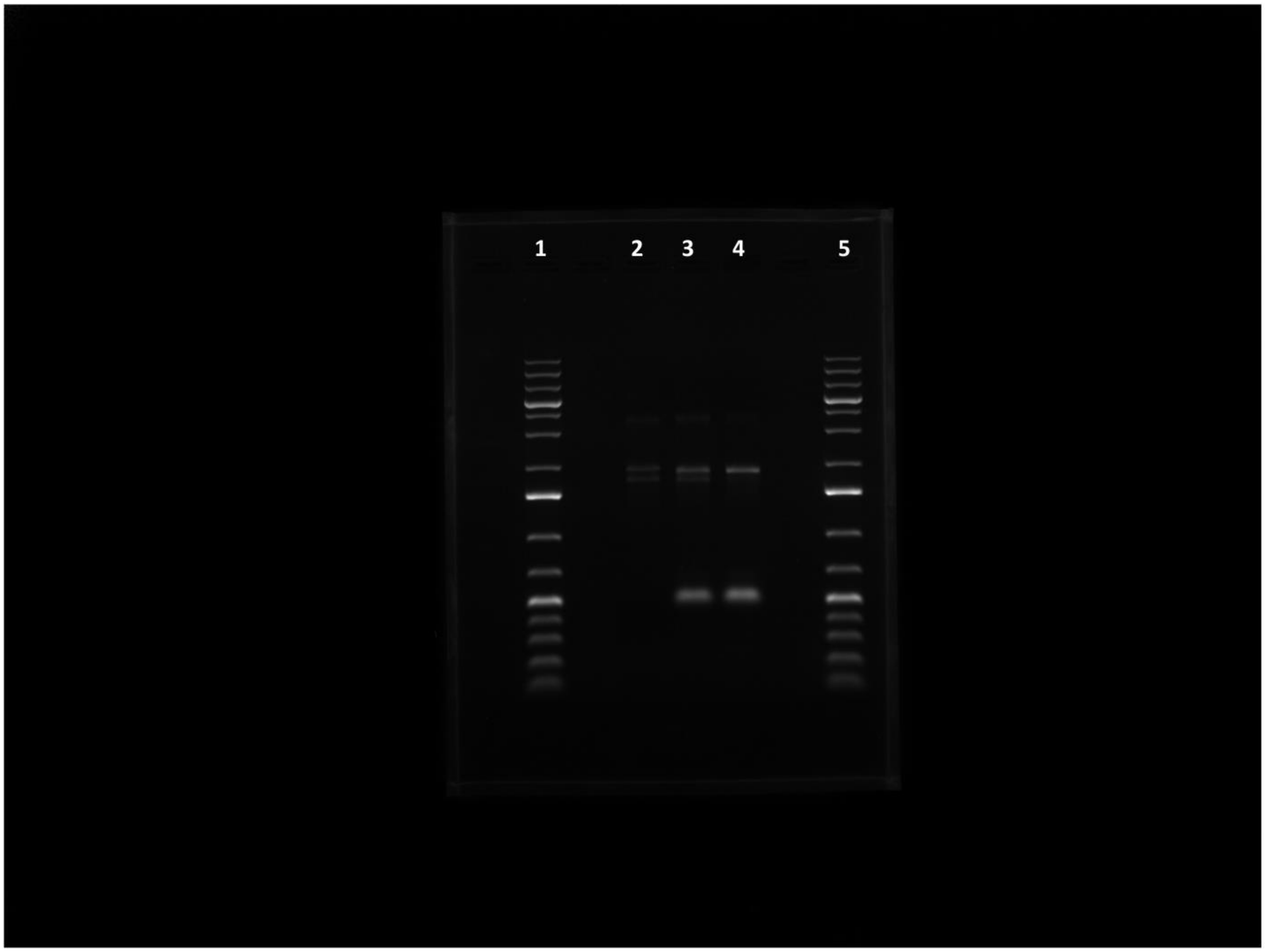


***Figure S9-4:*** *Unprocessed agarose gel image (0.7% agarose, 0.5× TBE, stained with 1× Gel Red, run at 70 V) used to create Figure S6 in the S6 Section of the Supporting Information, with loaded lanes enumerated. Full details of each loaded sample are available in the caption there (Figure S6, S6 Section).*
